# HMPV in Immunocompromised Patients: Frequency and Severity in Pediatric Oncology Patients

**DOI:** 10.3390/pathogens9010051

**Published:** 2020-01-10

**Authors:** Cesar Martinez-Rodriguez, Ma. del Rocio Banos-Lara

**Affiliations:** 1 School of Medicine. Instituto Universitario de Ciencias Médicas y Humanísticas de Nayarit; Tepic 63190, Mexico; martinezrodriguezcesar85@gmail.com; 2 Centro de Investigación Oncológica Una Nueva Esperanza-Universidad Popular Autónoma del Estado de Puebla; Universidad Popular Autónoma del Estado de Puebla, 21 sur #1103, Barrio de Santiago, Puebla 72410, Mexico

**Keywords:** pediatric cancer infections, HMPV, fatal cases in HSCT patients

## Abstract

Cancer is the first cause of death by disease in childhood globally. The most frequent types of cancers in children and adolescents are leukemias, followed by brain and central nervous system tumors and lymphomas. The recovery rate of cancer in children is around 80% in developed countries and up to 30% in developing countries. Some of the main causes of complications in children and adolescents with cancer are respiratory viral infections, mainly in bone marrow-transplanted patients. Respiratory viruses have been detected in the bronchoalveolar lavage or nasal wash specimens from cancer patients with or without respiratory illness symptoms. Human metapneumovirus (HMPV) is within the ten most common viruses that are encountered in samples from pediatric patients with underlying oncology conditions. In most of cases, HMPV is found as the only viral agent, but co-infection with other viruses or with bacterial agents has also been reported. The discrepancies between the most prevalent viral agents may be due to the different populations studied or the range of viral agents tested. Some of the cases of infection with HMPV in cancer patients have been fatal, especially in those who have received a hematopoietic stem cell transplant. This review seeks to show a general view of the participation of HMPV in respiratory illness as a complication of cancer in childhood and adolescence.

## 1. Childhood Cancer Global Situation

Cancer is the first cause of death by disease in children around the world. The five-year rate of survival of children who have cancer is up to 80% in developed countries, but for developing countries, the survival rate is less than 30% [1].

According to a study based in cancer registries from 52 countries, the types of cancer in childhood (0–19 years old) during 2001–2010 were leukemia, lymphoma, central nervous system tumors, sympathetic nervous system tumors, retinoblastoma, renal tumors, hepatic tumors, bone tumors, soft tissue sarcomas, germ cell and gonadal tumors, epithelial tumors, and melanomas. The most frequent cancers in children of 0–14 years are leukemia, followed by lymphoma; meanwhile, for the ages of 15–19, the most prevalent is lymphoma, followed by leukemia [2].

Cancer in childhood comprises only 1% of the total cancer cases worldwide; however, it has a dramatic impact. Recently the years of life lost due to cancer in childhood (0–19 years old) were calculated with global cancer registries in 2017, with a result of more than 11 million years [3].

## 2. Complications Due to Respiratory Viral Infection in Pediatric Oncology

The primary complications in pediatric cancer patients are mostly infections; in some cases, the risk of infection is the result of treatment-related neutropenia [4]. Furthermore, patients with pediatric cancer and those with hematopoietic stem cell transplantation are subjects to community-acquired respiratory virus infections, which are a cause of morbidity and mortality in this population. During the Fourth European Conference on Infections in Leukemia (ECIL-4), it was recommended to screen for respiratory viruses in patients with respiratory symptoms. The identification of influenza virus, human respiratory syncytial virus (HRSV), and human parainfluenza virus are the priorities. Meanwhile, the identification of human enterovirus, human metapneumovirus (HMPV), human rhinovirus, human coronavirus, and human adenovirus should be accomplished if the above-mentioned tests had negative results or when there is a risk of contagion [5]. 

HMPV is a leading cause of respiratory tract infections in children worldwide. Furthermore, HMPV is responsible for 7% of the respiratory diseases in adult patients with a hematological disease, as well as for the 7% in hematological stem cell transplant recipients [6]. 

## 3. Human Metapneumovirus

HMPV was isolated in 2001 from 28 children with symptomatology similar to that caused by HRSV; the more severe cases in that report were bronchiolitis and pneumonia [7]. Eighteen years after the description and characterization of HMPV, this virus was recognized as a leading cause of acute respiratory illness worldwide in infants, young children, the elderly, and patients with immunosuppression or other medical conditions [8]. The disease caused by HMPV is characterized by cough, fever, rhinorrhea, bronchitis, and bronchiolitis; in some cases, otalgia is also described by the patients [9].

HMPV is an enveloped virus with a negative-sense single-stranded RNA genome; HMPV isolates are classified phylogenetically into two major groups, A and B, which are subdivided into four subgroups, A1, A2, B1 and B2 [10].

## 4. Frequency of Human Metapneumovirus in Pediatric Patients

HMPV has been associated with 6.0–40.0% of acute respiratory illness in children, as was summarized by Shafagati and collaborators, with data from 2003 to 2013 [8]. Research recently performed in Belgium aimed at determining the frequency of HRSV and HMPV in samples from children and adults placed HMPV as the second most prevalent virus in respiratory samples, accounting for 7.3%, behind HRSV, which was encountered in 18% of the samples [11].

There have been studies with more extended search of viruses that have placed HMPV within the ten most prevalent viruses. In a study performed in Brazil, HMPV was found as the seventh most frequent causing agent of community-acquired pneumonia in children from 0.3 to 10 years old (12%) [12]; in the same way, analyses performed in China have situated HMPV as the eighth most frequent cause (5%) of severe acute respiratory infection in hospitalized children [13], the seventh most frequent cause (2.1%) of acute lower respiratory tract infection in children who were admitted to a pediatric intensive care unit [14], and the ninth (2.8%) most frequent virus in pediatric patients hospitalized with acute respiratory illness [15].

Patients going through an infection of HMPV usually have a favorable outcome; however, fatal cases have been reported in children without underlying medical conditions. Recently, two deaths due to HMPV infection were reported: the death of a 2.7 years old girl suffering from acute respiratory distress syndrome [16] and the death of a two-year-old old girl who suffered respiratory failure [17].

This review summarizes the cases of infection or death by HMPV in children and adolescents who have cancer as a basal medical condition.

## 5. Distribution of Respiratory Viruses in Pediatric Cancer

The distribution of respiratory viral pathogens in children and adolescents with cancer is summarized in Table 1. The types of cancer included in these studies were restricted to hematological malignancies and solid tumors, except for the work by Fazekas et al. [18], who additionally included inherited genetic disorders and primary immunodeficiencies in their study. The exact type of cancer of each study is shown in Table 2. Samples analyzed more frequently were nasopharyngeal aspirates, nasopharyngeal swabs, and bronchoalveolar lavages. 

Rhinovirus is the leading cause of respiratory diseases in oncology patients. It is detected in 10–62.7% of cases. Second to rhinovirus, the most represented virus is HRSV, found in 2.1–46% of cases. HMPV has been found in 3.3–13.5% of cases, being classified as the third [19], fourth [18,20,21,22,23], fifth [24] and tenth [25,26] most frequent respiratory virus, according to the literature reviewed. Interestingly, HMPV has been reported as the only viral agent that was responsible for an outbreak in a pediatric oncology population [27] (Table 1). 

**Table 1:** Viral findings in nasal samples from pediatric patients with respiratory illness and underlying oncology conditions.

Virus	Distribution of Viral Agents Found in Nasal Samples (%)										
Rhinovirus	33.1	10.0	37.0	30.8	36.5	36.8	--	23.1	62.7	22.7	--
Respiratory Syncytial Virus A/B	9.5	46.0	21.7	33.3	19.5	13.6	31.0	8.7	15.0	2.1	0.0
Metapneumovirus A/B	3.3	5.0	6.5	7.4	7.3	7.4	10.0	2.9	3.6	13.5	100
Coronavirus **(unspecified)**	--	1.0	8.7	--	19.5	--	--	--	11.4	--	--
Coronavirus 63	3.7	--	--	--	--	2.5	--	1.0	--	--	--
Coronavirus 229	3.7	--	--	24	--	1.2	--	2.9	--	--	--
Coronavirus 43	7.0	--	--	1.2	--	1.2	--	2.9	--	--	--
Coronavirus HKU	2.6	--	--	--	--	7.4	--	--	--	--	--
Cytomegalovirus			--	--	--					12.8	--
Influenza **(unspecified)**	--	--	--	--	9.7	--	36.0	--	--	--	0.0
Influenza A	--	2.0	2.2	11.1	--	11.1	--	1.9	8.8	14.2	--
Influenza A/H1N1	7.7	5.0	--	--	--	--	--	1.0	--	--	--
Influenza B	3.3	1.0	0.0	7.4	--	--	--	2.9	--	--	--
Influenza C	--	1.0	--	--	--	--	--	--	--	--	--
Parainfluenza (unspecified)	--	--	30.4	--	9.7	--	18.0	--	16.1	--	0.0
Parainfluenza 1	1.8	5.0	--	3.7	--	0	--	--	--	0.7	--
Parainfluenza 2	0.5	--	--	1.2	--	2.5	--	1.0	--	0	--
Parainfluenza 3	12.5	9.0	--	11.1	--	4.9	--	1.0	--	3.5	--
Parainfluenza 4	3.7	--	--	--	--	4.9	--	1.0	--	--	--
Bocavirus	4.8	13.0	0.0	--	2.4	2.5	--	--	7.8	2.8	--
Adenovirus	3.7	2.0	4.3	3.7	7.3	3.7	0.7	--	4.7	14.9	0.0
Enterovirus	1.2	--	--	--	4.8	11.1	--	--	0.5	--	--
Herpes simplex virus	--	--	--	--	--	--	--	--	--	--	--
Varicella zoster virus	--	--	--	--	--	--	--	--	--	--	--
Codetection of more than one virus	--	8.0	10.9	45.0	19.5	26.0	5.0	49.7	--	19.8	0.0
Samples	NA NPS	NPA G BAL	NPS	S	NS	TS	NPS NPW TA BAL	NPA	NPW TA BAL	TNA PB	NPA
Condition	ARI	URTI LRTI	RI	RI in ALL survi-vors	RI	ARI	ARI	ARI	URTI LRTI	LRTI and no RI	RI
Reference	[25]	[24]	[21]	[28]	[22]	[20]	[23]	[19]	[26]	[18]	[27]

Abbreviations. AS, anal swab; BAL, bronchoalveolar lavage; FN, febrile neutropenia; G, gargles; NA, nasal aspirate; NPA, nasopharyngeal swab; NPW, nasopharyngeal wash; NS, nasal swab; RI, respiratory illness; S, sputum; SS, skin swab; TA, tracheal aspirate; TS, throat swab; TNA, transnasal aspiration; PB, peripheral blood; ALL, acute lymphoblastic leukemia; ARI, acute respiratory infection; URTI, upper respiratory tract infection; LRTI, lower respiratory tract infection; and RI, respiratory tract infection.

## 6. Frequency of HMPV Infection in Pediatric Oncology 

Table 1 shows evidence of the place that HMPV takes in respect to other viral agents. However, there have been reports that only investigated the frequency of HMPV, and the most important information from these research works is condensed in Table 2.

We previously mentioned that HMPV has been found in 3.3–13.5% of acute respiratory infection (ARI) episodes (Table 1); nevertheless in febrile and neutropenia episodes, HMPV infection frequency has been found to be 0.4–44% (Table 2).

Co-infections were reported in most of the papers reviewed, but, in some cases, HMPV has been found as the only agent that caused respiratory illness in pediatric cancer patients, being 2.0–7.4% of the total virus distribution [25,28,29,30]. In some cases, bacteria or other pathogens have been detected along with HMPV [21,26,30,31,32] (Table 2).

Co-infections were described in detail in work by Torres and collaborators, who showed that HMPV is the sixth most frequent pathogen in sole infections, the fourth most frequent in mixed viral infections, and the sixth most frequent in virus–bacteria mixed infections [30].

**Table 2:** Human metapneumovirus (HMPV) frequency in pediatric oncology patients with respiratory illness or fever and neutropenia. When possible, only the information of cancer pediatric patients was obtained.

Underlying Pathology	Type of Study, Number of Patients, Number of Episodes and Age	Infections with Viral Etiology	HMPV Frequency	Co-infections with Other Viruses or Bacteria in HMPV Positive Samples	Fatal Cases with Viral Infection, or Deaths Attributable to Virus	Ref.
ALL, AML, RB, NHL, NB, rhabdomyosarcoma, JMML.	Prospective 118 patients; 81 patients with FN and ARI; 37 episodes with FN without ARI; inter quartile range 3.2–8 years; Median 4.5 years	80/118 (67.8%) had at least one virus	6/118 (5.1%)	21/118 (17.8%) with two or more viruses	1 case of influenza H1N1, *Klebsiella pneumoniae*, and *Acinetobacter* species; 1 case of HRSV; 1 case of adenovirus plus rhinovirus.	[20]
ALL, AML, Mixed ALL/AML, lymphoma, CNS tumor, NB, nephroblastoma, soft tissue sarcoma, bone tumors and HSCT	Prospective 224 patients; range 3–11 years; median 5 years	86/224 (38.4%) of cases had at least one virus	5/224 (2.2%)	18/224 (8.0%) with two or more viruses	5 cases of influenza, parainfluenza, and HRSV	[24]
ALL, AML, NHL, JMML, Mixed phenotype acute leukemia	Retrospective 74 patients; 93 episodes of respiratory illnesses; range 0–18 years; median 12 years	46/93 (49.4%)	3/93 (3.2%)	21/93 (22.6%) co-infection with bacteria and other pathogens. Co-infection with HMPV is not specified	No	[21]
ALL, AML, NHL, NB, sarcomas, nephroblastoma, Ewing sarcoma, osteosarcoma, hepatoblastoma, Burkitt lymphoma, HL, CNS tumor, LCH, and RB	Cross-sectional 108 patients; 219 episodes of URI; range 0.4–17 years; median 6.4 years	170 (77.6%) episodes had at least one virus	9/108 (3.3%)	None	No	[25]
ALL survivors after 6 months of therapy	Retrospective 54 patients; 23/54 without RVI and 31/54 with 81 episodes of RVI; range 7.2–18.1 years; median 11.2 years	31/54 (57%)	6/81 (7.4%)	No	No	[28]
AA, AML, ALL CML CLL. HL, NHL, MDS, MM, and solid tumors (unspecified)	Retrospective 90 patients; 181 ARI episodes; range 1–88 years; median 59 years	181 (100%)	181 (100%)	2 (1.1%) co-infected with *Stenotrophomonas maltophilia*, and *Aspergillus terreus*	12 cases positive for HMPV were fatal; 4 deaths were probably associated with HMPV; 2 of them were positive for *Stenotrophomonas maltophilia*, and *Aspergillus terreus*	[31]
Cancer (not specified)	Prospective 48 patients; 72 episodes of acute respiratory symptoms; range 0.6–18 years; median 8 years	41/72 (57%)	3/72 (5.5%)	1/72 (1.4%) adenovirus	No	[22]
ALL	Retrospective 223 patients; 133 ARI episodes; range 0–18 years; median NR	95/223 (42.6%) patients with ARI of viral etiology	13/133 (9.6%)	1/133 (2.2%) influenza A	No	[23]
ALL, AML, HL, NHL	Prospective 54 patients; 87 episodes of FN; range 0.5–17.7 years; median 7.0 years	39/87 (44.8%) With at least one virus	2/87 (2.2%)	No	No	[29]
Cancer (not specified)	Prospective 637 samples from patients with RTI; range 0.08–15.1 years; median 1.4 years (both for HMPV positive patients); 3 patients with cancer	637/637 (100%)	114/637 (17.9%) 88 patients 3/3 (100%)	39/88 (44.31%) with other viruses	No	[33]
Leukemia, lymphoma, and solid tumor (unspecified)	Prospective 525 patients; 1044 FN episodes; range 3–11 years; median NR	480/1044 (46%)	12/1044 (1.1%)	4/1044 (0.4%) rhinovirus 3/1044 (0.3%) HRSV 1/1044 (0.1%) rhinovirus and bocavirus	2 cases positive for rhinovirus and influenza	[34]
ALL, HR-NHL, AML, NHL, HR-NHL, HL, Sarcoma, CNS tumor, NB, nephroblastoma, LCH, CML, glioma, osteosarcoma, and hemophagocytic syndrome	Cross-sectional 48 patients; 104 fever and/or respiratory episodes; range NR; median 12 years	NR	3/104 (2.9%)	15/104 (14.4%) human rhinovirus	No	[19]
ALL, Wilms tumor, osteosarcoma, and ovarian cancer, HSCT	Retrospective 55 patients; range 0.4–19 y; median 5 years	55/55 (100%)	55 (100%)	3/55 (5%) 1/55 (2%) *Staphylococcus* 3/55 (7%) Other 3/55 (7%)	3 deaths for respiratory failure related to hMPV pneumonia; 2 of them were HSCT recipients, and they were treated with ribavirin and IVIG	[35]
Leukemia, lymphoma, solid tumor (unspecified,) HSCT, and brain tumor	Prospective 253 patients; 193 ARI episodes; range 0–18 years; median 6 years	193/253 (76.3%)	7/193 (3.6%)	1/253 (0.4%) rhinovirus, bocavirus, and *C. pneumoniae*	No	[26]
Leukemia, NB, rhabdomyosarcoma, hepatoblastoma, nasopharyngeal sarcoma, undifferentiated sarcoma, and HSCT	Retrospective 30 patients; range 0–18 years; median 4.5 years	NR	30/30 (100%)	6/30 (20%) *Staphylococcus* and *Streptococcus pneumonia* (detected in blood)	No	[32]
ALL, AML, NHL, histiocytosis, LMC, SD, Ewing sarcoma, soft tissue sarcoma, CNS tumor, system, hepatoblastoma, solid tumor (unspecified), rhabdoid intestinal tumor	Retrospective 35 patients; 37 ARI episodes; range 1–17 years; median 8 years	35/35 (100%) Acute lung injury	2/35 (5.7%)	1/35 (2.9%) HRSV	No	[36]
FN patients including leukemia, lymphoma, and solid tumor (not specified).	Prospective 190 episodes of FN; range 4–12 years; median 7 years	NR	14/190 (7.4%)	HMPV only infection 2.1%; co-infection with other viruses 3.6%; and co-infection with bacteria in 1.6%.	1 influenza A	[37]
ALL. AML HL, NHL, Ewing sarcoma, rhabdomyosarcoma, nephroblastoma, chronic leukemias, lymphomas, solid tumors, hematological diseases, inherited genetic disorders, and primary immunodeficiencies	Prospective 141 patients with or without LRTI with or without respiratory symptoms; range 0.4–17.6 years; mean 10.9 years	141/141(100%)	19/141 (13.5%)	5/141 (3.5%) co-infection with HRSV, influenza A, bocavirus, rhinovirus, or cytomegalovirus	No	[18]
AML, hemophagocytic lymphohistiocytosis among other hemato-oncological diseases not specified	Retrospective 15 patients; range 0.3–7 years; median 1.6 years	15/15 (100%)	15/15 (100%)	No	No	[27]
ALL	Prospective 51 patients; 138 febrile episodes; range 0.4–15.2 years; mean 5.9 years	NR	61/138 (44.2%)	12/138 (8.6%)	2 rhinovirus and *Aspergillus*; rhinovirus and *Streptococcus mitis*	[37]
Hematological cancer and solid tumors (not specified)	Prospective 66 patients; 250 febrile episodes; Pediatric age (unspecified)	19/250 (7.6%)	1/250 (0.4%)	None	1 (5.5%) death for HRSV pneumonia	[38]
AML, ALL, CML, CLL, MDS Lymphoma MM, NB, Wiskott–Aldrich Syndrome, SCID, Fanconi anemia, Amyloidosis, Solid tumor (Breast, genitourinary, brain, lung and others) and patients with HSCT	Retrospective and prospective 1899 patients; range NR median 35.9 years; 37% patients were ≤21 years	NR	51/1899 (2.7%)	NR	None	[39]
ALL	Retrospective 2 patients with ALL out of 12 pediatric patients hospitalized for respiratory conditions <5 years; median 1.3 years	2/2 (100%)	2/2 (100%)	NR	1 patient with ALL died of ARDS probably due to HMPV	[40]
Head and neck Burkitt lymphoma	Case report 1 patient of 2 years	NR	One case	No	No patient was treated with ribavirin and IVIG	[41]
Burkitt lymphoma	Case report 1 patient of 8 years	NR	One case	No	No patient was treated with ribavirin and IVIG	[42]
ALL	Case report 1 patient of 4 years	NR	One case	No	No patient was treated with ribavirin and IVIG	[43]
ALL	Case report 1 patient of 0.6 years	--	One case	No	Patient died probably for HMPV	[44]

Abbreviations. AA, aplastic anemia; ALL, acute lymphoblastic leukemia; AML, acute myeloid leukemia; ARI, acute respiratory infection; ARDS, acute respiratory distress syndrome; CML, chronic myeloid leukemia; CNS, central nervous system; FN, fever and neutropenia; HL, Hodgkin’s lymphoma; HSCT, hematopoietic stem cell transplantation; HR-ALL, high-risk acute lymphoblastic leukemia; HR-NHL, high-risk non-Hodgkin’s lymphoma; IVIG, intravenous immunoglobulin; JMML; juvenile myelomonocytic leukemia; LCH, Langerhans cell histiocytosis; MDS, myelodysplastic syndrome; MM, multiple myeloma; NB, neuroblastoma NHL; non-Hodgkin’s lymphoma; NR, not reported; and RB, retinoblastoma.

## 7. Fatal Cases Due to HMPV Infection in Pediatric Patients with an Underlying Oncology Condition 

Fatal cases in pediatric patients with no underlying medical condition due to HMPV are known [17,45]; HMPV has also been related to a fatal case of encephalitis [46]. Though rare, some fatal cases due to HMPV infection have been reported for pediatric cancer patients (Table 2) and for those who are recipients of a hematological cell transplant (Table 3).

As a result of this review, we found 17 cases of death that were positive for HMPV; in nine cases, HMPV was the only pathogen found [31,35,40,44]. However, some of these investigations considered children and adults [31,39] or children without cancer [33]. Excluding these investigations, we counted 3233 cases of children and adolescents with cancer who showed a respiratory disease or fever and neutropenia episodes. Out of the 3233 cases, 227 children were positive for HMPV (7%). Three deaths were reported, which means that 0.09% (3/3233) of the total patients succumbed and 1.3% (3/227) of the patients infected with HMPV succumbed (Table 2). 

It is important to mention that at least in one piece of research, the authors tested HMPV in pediatric hematopoietic stem cell transplant patients, but they found nothing [47].

Regarding patients who had received a hematopoietic cell transplant (Table 3), we counted 128 infections with HMPV in 2262 patients (5.6%), and three fatal cases. This means that 5.6% (128/2262) had an HMPV infection, 0.13% (3/2262) of the patients with a transplant that was infected with HMPV died, and 2.3% (3/128) of the patients positive for HMPV died (Table 3).

Of note, three out of the five patients who were treated with ribavirin plus intravenous immunoglobulin survived [41,42,43]; the other two did not [35]. The mentioned treatment has also been proven to reduce the risk of mortality for HRSV in hematopoietic stem cell-transplanted patients [48].

Cases of death in adult patients who have received a transplant of hematopoietic stem cells due to onco-hematological conditions, ascribable undoubtfully or presumably to HMPV, have been reported [49,50,51,52]. A systematic review by Shah and collaborators gathered 4208 cases of hematologic malignancy in children and adults who received a hematopoietic cell transplant. In that review, they showed that 34% of the analyzed cases of lower respiratory tract infection (LRTI) were due to HMPV, and 6% were fatal [6].

**Table 3:** Distribution of HMPV infection in hematopoietic stem cell transplantation (HSCT) patients after an oncology condition.

Transplant and Underlying Condition	Type of Study, Number of Patients, and Age	Infections with Viral Etiology	hMPV Frequency/Fatal Cases Due to HMPV Infection	Reference
HSCT. **Primary immune deficiency pathologies, leukemia, lymphoma, CNS tumor, solid tumor.**	Retrospective study 39 cases of HMPV infection; range 0–18 years; median 6 years	39 cases of HMPV infection	39/39 (100%) no fatal cases	[53]
HSCT. **ALL AML, MDS,** SAA, **inherited marrow failure, primary immune deficiency**, **NHL, Hodgkin’s disease**, **CML, metabolic diseases, hemophagocytic lymphohistiocytosis, brain tumors, solid tumors,** and **thalassemia.**	Retrospective study 844 HSCT cases; range 0.1–17.8 years; median 7.5 years	96 respiratory viral infection	2/844 (0.1%) no fatal cases	[54]
HCT. Congenital hematologic disorders, congenital immunodeficiency diseases, paroxysmal nocturnal hemoglobinuria, AA, CML, myelodysplastic syndromes, leukemia and lymphoma, and other hematologic malignancies.	Retrospective and prospective 458 patients with HCT virus were determined before and after HCT 52 pediatric patients <18 years	Positive for virus after 100 days of HCT 27/342 (7.8%); originally negative, and 18/116 (15.5%) originally positive.	1 fatal case—2 years old patient with AA	[55]
HSCT. Hematological disease (unspecified)	Prospective 378 patients; range 0.1–17.8 years; median 7.5 years	166 VRI in 131 out of 378 patients (34.6%)	12/378 (3.17%) No fatal cases	[56]
HSCT. **AA, SCID, Ewing sarcoma ALL and osteopetrosis**	Retrospective 55 patients; range 0.4–19 years; median 5 years	55 cases of HMPV infection	55/55 (100%) 2 fatal cases patients were treated with ribavirin and IVIG	[35]
HSCT. ALD, SAA, PNH, ALL	Retrospective 769 patients; range 3–41 years; mean 18.3 years	186 (24.6%)	19/769 (2.5%) no fatal cases	[57]
HSCT CML	Case report 10 years	1	1 fatal case	[58]
Autologous HSCT NB. MB, ependymoma, RB, primitive neuroectodermal tumor, Wilms tumor, atypical teratoid/rhabdoid tumor, germ cell tumor, osteosarcoma, T lymphoblastic lymphoma, diffuse large B-cell lymphoma, Ewing sarcoma, malignant fibrous histiocytoma, NHL, lymphoma, Yolk sac tumor. Allogenic HSCT AML, ALL, MPAL, JMML, AA, hemophagocytic lymphohistiocytosis, NB, B Wiskott–Aldrich syndrome, HL T-cell/histiocyte-rich large B-cell lymphoma, Fanconi anemia, MB, and myeloid sarcoma	Retrospective 176 HSCT recipients, Autologous HSCT range 0–19 years; mean 4 years Allogenic HSCT range 0–17 years; mean 6 years	84/176 (47.7%) with respiratory symptoms	No fatal cases	[47]

Abbreviations. AA, aplastic anemia; ALL, acute lymphoblastic leukemia; ALD, adrenoleukodystrophy; AML, acute myeloid leukemia; CML, chronic myeloid leukemia; CNS, central nervous system; HL, Hodgkin’s lymphoma; HCT, hematopoietic cell transplantation; HSCT, hematopoietic stem cell transplantation; IVIG, intravenous immunoglobulin; PNH, paroxysmal nocturnal hemoglobinuria, MDS, myelodysplastic syndrome; MPAL, mixed phenotype acute leukemia; JMML, juvenile myelomonocytic leukemia; MB, medulloblastoma; MM, multiple myeloma; NB, neuroblastoma; NHL, non-Hodgkin’s lymphoma; SCID, severe combined immunodeficiency; SAA, severe aplastic anemia; NR, not reported; and RB, retinoblastoma.

## 8. Conclusions

Pediatric oncology patients are more susceptible infections due, in most cases, to the neutropenia that results from medication.

HMPV infections in patients with hematological conditions have been documented, but their impact in childhood is not clear because the research has involved children and adults or only adults. This review shows the respiratory viral infections that can be originated to HMPV infection exclusively in pediatric oncology patients. The distribution of HMPV among other viruses in otherwise healthy children and adolescents is reported to be 6–40% [8], which is greater than that of the pediatric population with underlying cancer, whose HMPV distribution is 3.3–13.5% (as seen in this review). These data should be taken carefully because the combined number of patients in the cancer group was small (n = 3867), and the number of HMPV infections may have been underestimated. Interestingly, in pediatric oncology patients, 2.9–10% of the HMPV infections were detected in episodes of ARI; however in children going through febrile episodes, HMPV infection was found in up to 44% [37] and 51% [38] of cases. This difference is probably because HMPV infections could be asymptomatic because about 60% of the fever episodes in neutropenic patients do not show other signs or symptoms [59]. 

HMPV has become more relevant because outbreaks of infection in pediatric [27] and adult cancer patients [60] have occurred with fatalities. Some deaths attributable to HMPV infection have been reported in the pediatric oncology population, with and without a hematopoietic stem cell transplant [31,35,40,44,55,58]. According to the analyzed information, 1.3% of pediatric patients with cancer have been found to be positive for an HMPV infection and had a fatal outcome; meanwhile, 2.3% of the pediatric cancer patients who were recipients of a transplant have been found to be positive for HMPV had death. 

The management of patients with HMPV is mainly supportive. The treatment of two patients with ribavirin and intravenous immunoglobulin in three cases of Burkitt lymphoma [41,42] and one case of acute lymphoblastic leukemia (ALL) [43] has been to be effective against infection with HMPV; this treatment has also been shown to be effective in the cases of two adults with ribavirin following hematopoietic stem cell transplantation (HSCT) [61] and lung transplant [62]. Though the previously described treatment has been proved to be effective, no randomized clinical trials have been performed to support these results. Actually, the treatment with ribavirin and intravenous immunoglobulin (IVIG) had an unfavorable outcome in other cases [35]. The lack of an approved treatment for HMPV infection has increased the possibility of fatal outcomes in a population at risk, and so research to find efficient treatments or vaccines for HMPV should be encouraged. 

It is important to note that HMPV infections are frequently accompanied by other viral or bacterial infections. One third of the children with cancer who present viral infections are co-infected with bacteria, and children with viral infections stay for fewer days in the hospital than children with mixed virus–bacterial infections [30]. The question of where HMPV infections contribute to the progress of other infections needs to be explored, especially in immunodeficient children.

Research regarding HMPV infections exclusively in the pediatric cancer population is available, but the estimations made from this research have limitations, mainly due to the number of analyzed patients. Thus, more work in this field is imperative to have a better understanding of the impact of HMPV and other respiratory viruses in populations at risk.

## 9. Methodology

Two researchers (C.M.R. and M.R.B.L) independently performed a search in PubMed with different combinations of the terms HMPV, metapneumovirus, and respiratory viral infection, as well as cancer children, leukemia children, and pediatric oncology. As a result, a total of 811 papers were found. A second search with the terms HMPV, metapneumovirus, and transplant cancer retrieved 61 papers. After reading the abstracts, reviews and duplicates were eliminated. Papers in which the subjects of the studies were only adults and those in which HMPV was not surveyed were also eliminated. Papers written in English and Spanish were all considered. In the end, the number of papers that met the established characteristics was 30. During the process of writing of the review, four more papers were included.

Each researcher designed different tables for gathering the most critical data out of the papers while trying to keep as much common information as possible. Afterwards, the researchers shared and compared their found information; as a result, three tables were obtained.
